# Incorporating patient preferences in the management of multiple long-term conditions: is this a role for clinical practice guidelines?

**DOI:** 10.15256/joc.2015.5.53

**Published:** 2015-11-11

**Authors:** Charlotte E. Young, Frances M. Boyle, Katie S. Brooker, Allyson J. Mutch

**Affiliations:** ^1^School of Public Health, Faculty of Medicine and Biomedical Sciences, The University of Queensland, Brisbane, QLD, Australia; ^2^Queensland Centre for Intellectual and Developmental Disability (QCIDD), School of Medicine, Mater Research Institute, The University of Queensland, Brisbane, QLD, Australia

**Keywords:** multimorbidity, comorbidity, primary care, patient preference, consumer participation, clinical practice guidelines

## Abstract

**Background:**

Clinical practice guidelines provide an evidence-based approach to managing single chronic conditions, but their applicability to multiple conditions has been actively debated. Incorporating patient-preference recommendations and involving consumers in guideline development may enhance their applicability, but further understanding is needed.

**Objectives:**

To assess guidelines that include recommendations for comorbid conditions to determine the extent to which they incorporate patient-preference recommendations; use consumer-engagement processes during development, and, if so, whether these processes produce more patient-preference recommendations; and meet standard quality criteria, particularly in relation to stakeholder involvement.

**Design:**

A review of Australian guidelines published from 2006 to 2014 that incorporated recommendations for managing comorbid conditions in primary care. Document analysis of guidelines examined the presence of patient-preference recommendations and the consumer-engagement processes used. The Appraisal of Guidelines for Research and Evaluation instrument was used to assess guideline quality.

**Results:**

Thirteen guidelines were reviewed. Twelve included at least one core patient-preference recommendation. Ten used consumer-engagement processes, including participation in development groups (seven guidelines) and reviewing drafts (ten guidelines). More extensive consumer engagement was generally linked to greater incorporation of patient-preference recommendations. Overall quality of guidelines was mixed, particularly in relation to stakeholder involvement.

**Conclusions:**

Guidelines do incorporate some patient-preference recommendations, but more explicit acknowledgement is required. Consumer-engagement processes used during guideline development have the potential to assist in identifying patient preferences, but further research is needed. Clarification of the consumer role and investment in consumer training may strengthen these processes.

## Introduction

Clinical practice guidelines targeting specific long-term conditions provide an evidence-based approach to treatment and management and can lead to improved patient care [1]. However, the ability of guidelines to support complex care regimes for patients with multiple long-term conditions is the subject of some debate [2–6]. The use of multiple disease-specific guidelines for individual patients is impractical and potentially hazardous [2–5]. Alternative approaches to addressing multiple conditions using clinical practice guidelines have been proposed and considered [4, 7]. These include the development of meta-guidelines, which address common clusters of co-occurring conditions [7] and greater cross-referencing between guidelines that are available electronically [4]. Some single-condition-specific guidelines include recommendations addressing comorbid conditions [3]; but the extent to which these guidelines also consider patient preferences is unclear.

While the debate about the adaption of clinical guidelines continues, a strong theme in the literature is the need to foster a patient-centred approach to the management of multimorbidities and take greater account of what patients want and value [8–11]. Patient preferences – “*the desirability of a health-related outcome, process or treatment choice*” [10] – are considered important for the management of multiple and competing health conditions as the patient’s focus is shifted from disease-specific goals to more global cross-disease outcomes, such as maintenance of physical function, symptom relief and quality of life [12, 13]. In essence, some recommendations may be acceptable to most patients, but others may be “preference-sensitive” and dependent on the patient’s views about outcome, process or choice [10]. Researchers argue that incorporating patient preferences may mitigate the common criticism that guidelines developed to address single conditions are created for the “average patient” and do not acknowledge the complexity of individuals’ circumstances and preferences [8, 14].

Krahn and Naglie [10] argue that the identification and incorporation of patient preferences in guideline development and implementation may improve the patient-centeredness of clinical practice guidelines. They suggest that obtaining consumer input during guideline development may provide the foundation for greater systematic attention to patient preferences and support for patient decision-making in clinical consultations [10, 15, 16]. Strategies to engage consumers in guideline development include providing drafts for feedback, involving consumers in guideline-development groups, conducting surveys of consumers or running consumer focus groups or workshops parallel to the clinical guideline development groups [9, 17–19]. Some of these approaches have been criticized for being passive or “tokenistic” [18, 19], but broader evidence assessing their impact on guideline development is limited, with the exception of a recent study by Tong *et al*. [16], which found that active consumer engagement led to the identification of patient-centred recommendations not flagged by health professionals.

The widely used Appraisal of Guidelines for Research and Evaluation (AGREE) Instrument [20] acknowledges the importance of consumer input by way of an item assessing whether the views and preferences of the target population (patients, public, etc.) have been sought. In ­Australia, for clinical practice guidelines to receive approval from the National Health and Medical Research Council (NHMRC) [21], they must “*be developed by a multidisciplinary group that includes relevant experts, end users and consumers affected by the clinical practice guideline*”. While consumer engagement is strongly advocated, it is unclear how such engagement takes place or whether it leads to greater inclusion of patient-­preference recommendations in clinical guidelines. More broadly, the extent to which clinical practice guidelines encourage patient-centred care through the inclusion of patient preferences also requires further investigation.

## Objectives

The objectives of this study were to review clinical practice guidelines that include recommendations for comorbid conditions to determine the extent to which they: (1) incorporate patient-preference recommendations; (2) use consumer-engagement processes in their development phase; and (3) meet standard criteria for guideline quality, particularly in relation to the stakeholder-involvement processes; and to consider whether consumer-engagement processes in guideline development result in greater integration of patient-preference recommendations.

## Methods

### Inclusion criteria

The study examined Australian clinical practice guidelines developed to support single chronic conditions, but which included recommendations for comorbid conditions (i.e. medical conditions additional to the index condition [22]).

All guidelines developed to support the National Health Priority areas were included: ­cardiovascular health; stroke; cancer (colorectal, lung, breast and prostate); diabetes; depression; chronic kidney disease; asthma and chronic obstructive pulmonary disease; and arthritis and musculoskeletal conditions [23]. Additional selection criteria included: application in primary care settings; and applied to people aged 18 years and over. Guidelines are updated approximately every 6 years; therefore the search, which began in 2012, focused on guidelines published between 2006 and 2012. The search was later extended to include publications up to January 2014.

### Search strategy

Ovid MEDLINE, Web of Science (ISI), Embase, Cinahl, PsycINFO, Cochrane and PubMed, were searched using the terms: “guideline”, “Australia”, and “primary care”. Additional searches were conducted on Australian websites, including the Department of Health, NHMRC, National Institute of Clinical Studies, Royal Australian College of General Practitioners and relevant non-profit organization websites. The Medical Journal of Australia and the Internal Medicine Journal, key journals publishing clinical guidelines, were also searched.

### Study selection

**Figure 1:**
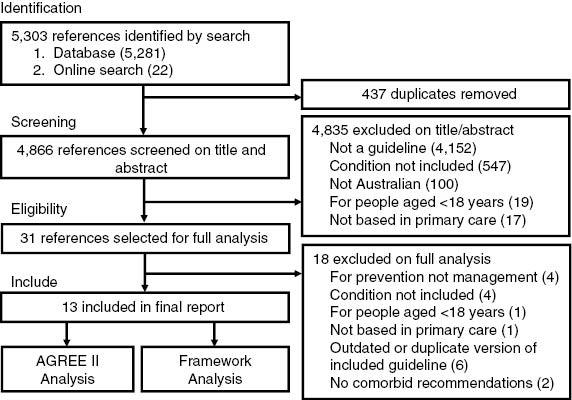
Search strategy. AGREE II, Appraisal of Guidelines for Research and Evaluation II.

Figure 1 summarizes the guideline selection process. In all, 4,866 citations were identified: 4,835 of these were excluded, based on title and summary. The full text of 31 guidelines was reviewed. Eighteen were excluded because they: did not provide recommendations for comorbid conditions; focused on prevention and detection; addressed out-of-scope conditions; targeted young people; were not applicable to primary care; or were outdated versions of an included guideline. Clinical updates or addenda were assessed in conjunction with the original guideline. Thirteen guidelines were included in the final analysis.

### Data analysis

Data analysis was conducted in three stages in accordance with the three main aims of the study. Stages 1 and 2 involved document analysis and Stage 3 involved a quality assessment using AGREE II. Ethics approval was not required as all data were drawn from published materials available in the public domain.

#### Stage 1

Document analysis of the guidelines was conducted to identify recommendations that incorporated patient preferences. Clinical practice guidelines vary in complexity and size, ranging in length from ten to several hundred pages and frequently provide a list of core recommendations or essential points, which are then further explained throughout the document by “supporting evidence statements”. The core recommendations and supporting evidence statements were analysed to identify recommendations that focused on patient preferences.

Analysis was directed by the framework approach [24], which involved five steps (familiarization; identifying a thematic framework; indexing; charting; and mapping and interpretation). Detailed review of the guidelines ensured familiarity with content and enabled the identification of key themes that aligned with the notion of patient preferences. An index framework that defined key themes including and consistent with patient preferences (e.g. “actively involved” recommended patients be engaged, involved or given the opportunity to participate in the decision-making process) was developed and used to code content. Relevant passages from each guideline were extracted in accordance with the themes and placed in charts to assist with mapping and interpreting the data.

Three guidelines provided recommendations and evidence statements for both children and adults [25–27]. Recommendations and evidence statements that focused only on children were excluded from the analysis.

#### Stage 2

Document analysis using the framework approach was also conducted to assess the consumer-engagement processes used during guideline development. Explanation of guideline-development processes, including consumer engagement, was typically described at the beginning or end of the guideline, or occasionally in a separate report. All of this material was reviewed. A thematic framework was developed from key themes identified in the document analysis and from the literature (e.g. “training and education”). This framework was used to code guideline content.

#### Stage 3

An assessment of guideline quality was conducted using the AGREE II instrument [20]. AGREE II is a validated tool that assesses guideline quality according to 23 items listed under six domains: scope and purpose; stakeholder involvement; rigour of development; clarity of presentation; applicability; and editorial independence [5]. For each domain, questions are scored on a 7-point scale from 7 (strongly agree) to 1 (strongly disagree). An overall domain score was calculated from the sum of individual items standardized as a percentage for each domain [20]. Guidelines were assessed as “good quality” [3] if they scored above 60% on all of the AGREE II domains. Two reviewers (C.E.Y. and K.S.B.) independently scored each guideline. The AGREE II concordance calculator [28] confirmed an acceptable level of agreement between the reviewers.

In line with the study aims to examine consumer-engagement processes, particular attention was paid to the stakeholder domain in the AGREE II, which includes three items: 1) guideline development includes individuals from all relevant professional groups; 2) the views and preferences of the target population (e.g. patients, public) have been sought; and 3) the target users of the guideline are clearly defined [20].

## Results

Thirteen guidelines met the inclusion criteria: four guidelines for cardiovascular health [29–32]; one guideline for stroke [33]; one guideline for prostate cancer [34]; two guidelines for diabetes mellitus [25, 26]; two guidelines for musculoskeletal health [35, 36]; two guidelines for respiratory conditions [27, 37]; and one guideline targeted multiple chronic conditions focusing on the prevention and management of chronic kidney disease for people with type 2 diabetes [38]. Eleven guidelines were developed by non-profit organizations [25–27, 29–34, 37, 38] and two guidelines by the Royal Australian College of General Practitioners [35, 36]. Five guidelines were approved by the NHMRC [25, 33, 35, 36, 38]. The guidelines ranged in length from 19 to 288 pages.

### Incorporating patient preferences

Across the 13 guidelines, a total of 1,076 core recommendations were reviewed, of which 49 (4.5%) were identified as patient-preference-related recommendations (see Table 1). The number of total core recommendations ranged from 18 to 335 for individual guidelines and the number of core patient-preference-related recommendations ranged from 0 to 16 (0–12.2% of the total core recommendations). A further 108 statements, directing clinicians to consider patient preferences, were identified in the supporting evidence statements (range 0–25).

Examination of both the guideline recommendations and supporting evidence statements revealed four key themes: patient preferences; care plans; actively involved; and risks and benefits (Table 1). General introductory comments or “blanket statements” [8] emphasizing the need to consider individuals’ views were also identified.

Twelve guidelines explicitly asked for patient preferences to be considered by the clinician in relation to treatment, interventions, or outcomes [25–27, 29–37]. For example, the guideline on type 1 diabetes stated: *“Choice of device should be made on the basis of ease of use, patient preference/suitability and overall cost”* [25]*.*

Care plans, also referred to as management, action and treatment plans, were highlighted by all but two guidelines [32, 38] as a means of working collaboratively with patients to identify their preferences and goals for care. Care plans were the most frequently flagged core patient-preference-related recommendations (range 0–13) and were also commonly discussed in the supporting evidence statements (range 2–6) (see Table 1). For example, the guideline for rheumatoid arthritis stated the following:* “General practitioners should aim to engage patients with RA [rheumatoid arthritis] in individualised care plans that include treatment goals and objective measures of disease”* [35]*.*

Seven guidelines [25–27, 32, 33, 35, 37] called for patients to be actively involved or engaged in decision-making and as a member of the healthcare team, as illustrated by this example from the guideline for type 2 diabetes: *“Encourage patients to participate and take an active role in the management of their diabetes”* [26]*.*

Five guidelines [27, 31, 33, 34, 36] suggested outlining the risks and benefits of recommended treatments to enable patients to make an informed decision based on their treatment preferences, as demonstrated by the guideline for prostate cancer: *“Toxicities should be considered in the context of what is important to each individual patient, as for some patients impairment of sexual function may have a significant impact on their quality of life and overall adjustment, as well as affecting adversely those close to them”* [34]*.*

Eleven guidelines provided blanket statements [25–27, 30–36, 38]. These were statements provided at the beginning of the document, instructing clinicians that all recommendations should be individualized to consider the needs, preferences and context of each patient. For example, the chronic kidney disease in type 2 diabetes guideline, the only guideline that did not include any core patient-preference-related recommendations, began with the following overarching statement: *“This document is a general guide to appropriate practice, to be followed subject to the clinician’s judgement and the patient’s preference in each individual case. The guidelines are designed to provide information to assist decision-making and are based on the best evidence available at the time of development”* [38].

### Guideline quality

The AGREE II domain scores for each guideline are presented in Table 2. Across all guidelines, the *applicability* domain (i.e. “*has the guideline outlined potential barriers and facilitators to its implementation in practice, strategies to improve uptake, and resource implications*”) received the lowest domain scores, while *clarity of presentation* (i.e. “*are the recommendations specific and easily identifiable, and are the various options clearly presented*”) received the highest domain scores [20]. Clarity of presentation was the only domain for which all guidelines scored above 60%.

The five guidelines approved by the NHMRC consistently scored higher across all domains [25, 33, 35, 36, 38]. Within this group, the guideline for stroke was the only guideline to score above 60% in all domains [33].

Of particular interest was the *stakeholder involvement* domain. Six guidelines scored above 60% for this domain [25, 33–36, 38]. Closer examination of the individual items within this domain revealed that 11 guidelines scored better (average between the two reviewers above 4.2 (60%) on a 7-point rating scale) for the first item: “*included individuals from all relevant professional groups in the development group*” [25, 27, 29, 30, 32–38]; and the third item: “*clearly defined target users*” [25–27, 30–36, 38]. In contrast, only four guidelines scored above 60% [25, 33, 34, 38] on the second item “*the views and preferences of the target population (patients, public, etc.) have been sought*”. To score highly on this item, guideline developers needed to outline the strategies used to gain consumer perspectives, report the outcomes of this process and describe how this was used to inform the guideline.

### Consumer-engagement processes

The thematic framework developed for this phase of the analysis covered four key themes: consumer involvement in the development group; clarification of this role; provision of drafts for public review; and training and education. Across the guidelines, only two methods of engagement were reported: involving consumer representative(s) in guideline-development groups; and providing drafts for public review. Seven guidelines used both methods [25, 33–38], three provided drafts for public review only [29, 31, 32], and three did not report their methods of engagement [26, 27, 30] (see Table 3).

Most guideline-development groups included one or two consumer representatives recruited from relevant non-profit organizations, with the exception of the guideline for stroke [33], which included three consumers, and the guideline for chronic kidney disease in type 2 diabetes [38], which included five consumers, one for each of the five smaller expert advisory groups forming the guideline-development group (see Table 3). None of the guidelines reported training and education of consumers. Seven guidelines reported the broad role of the development group [25, 33–38]; only one provided a specific explanation of the consumer’s role [38]. *“Consumer representatives were selected and appointed by Diabetes Australia for each EAG [Expert Advisory Group] to ensure the consideration of people with type 2 diabetes with respect to their acceptability of the proposed guideline recommendations”* [38].

Ten guidelines provided drafts for public review [25, 29, 31–38], but the extent to which this engaged consumers was not always possible to ascertain. Four guidelines provided an explanation of the results of the public-review processes, outlining how comments were incorporated or changes made [25, 33, 34, 38]. The comments addressed a range of issues including guideline structure, chapter size, editing, and clarification of recommendations and supporting evidence statements. Six guidelines did not clarify the extent or nature of the feedback process [29, 31, 32, 35–37]. For example, the guideline on osteoarthritis stated:* “Feedback collected from the survey and independent submissions were collated and addressed by the Working Group”* [36].

The study also considered whether consumer-engagement processes in guideline development resulted in greater integration of patient preferences. There was some suggestion that more extensive use of consumer-engagement processes (i.e. both provision of drafts for public review and inclusion of consumers in the development group) was associated with greater incorporation of patient-preference recommendations. Six of the seven guidelines that produced the greatest proportion of core patient-preference recommendations (as shown in Table 1) used both consumer-engagement methods [25, 33–37]. However, the guideline that reported the most comprehensive consumer-engagement processes (i.e. provided a specific explanation of the consumers’ role, included five consumers in the development group, and stated how the public review feedback was incorporated) [38] did not explicitly include recommendations targeting patient preferences. Rather a blanket statement was provided at the beginning of the document calling for patient preferences to be considered when applying the guideline to individual patients.

## Discussion

All 13 of the reviewed guidelines acknowledged patient preferences, either explicitly or indirectly through related themes [25–27, 29–38], but their location and prominence varied, appearing as core recommendations, supporting evidence statements and/or blanket statements. Ten guidelines reported some form of consumer engagement during their development [25, 29, 31–38]. The guidelines that employed the most extensive consumer-engagement processes (e.g. both provision of drafts for public review and inclusion of consumers in the development group) were among those with the greatest proportion of patient-preference recommendations [25, 33–38]. Overall, the quality of guidelines was mixed; the lack of evidence of strategies to incorporate the views and preferences of consumers saw many fall short on stakeholder involvement.

Quantifying the extent to which guidelines incorporate patient preferences was not always straightforward, as some recommendations and supporting evidence statements were less explicit in their request that patient preferences be considered. Similarly, patient preferences were more frequently presented in supporting evidence statements than in core recommendations. Presenting patient preference information in supporting evidence statements may undermine the potential of guidelines to support a more systematic discussion of patient preferences in primary care as it risks this information being overlooked by time-poor clinicians. In practice, clinicians and patients frequently identify differences in their preferences, priorities and goals for care when managing multiple conditions; if not discussed and worked through, these differences can lead patients to disengage from clinical advice [39]. Clearer and more frequent flagging of patient-preference-related recommendations in guidelines is needed to draw attention to patient preferences in clinical consultations. Consistently identifying these recommendations and facilitating their discussion is one way in which guidelines might support a more systematic approach to patient-centred care [10, 16].

Overall, more extensive use of consumer-engagement processes in guideline development was linked to a greater proportion of core patient-preference recommendations; however, closer consideration of the consumer-engagement processes used across the guidelines highlights shortfalls in practice. Ten guidelines engaged consumers in public-review processes [25, 29, 31–38]. These typically ‘passive’ methods have been criticized for limiting consumers’ ability to actively engage and provide valuable input [16, 19, 40]. Seven guidelines engaged consumers in development groups [25, 33–38], but there was little evidence of the provision of training or specific role descriptions for consumers.

Research suggests that unless guideline developers provide consumers participating in development groups with education and training, a clear explanation of their role, and sufficient support (e.g. more than one consumer representative), their involvement is likely to be tokenistic and relatively ineffective [17–19]. In support, Tong *et al*. [16] found that, when adequately assisted and engaged, consumers were able to contribute meaningfully to guideline development by identifying topics and outcomes (e.g. day-to-day management and overall illness experience) not identified by health professionals. This experiential input is the cornerstone of consumer engagement: it extends the clinicians’ focus from disease to incorporate the patients’ social context, experiences, and feelings [14]. In short, without effectively engaging consumers, guideline developers risk producing guidelines that may not fully address the topics and outcomes of importance to patients, particularly those experiencing multiple conditions [14, 16, 19].

Our findings, like those of Vitry and Zhang [6], demonstrate the role of NHMRC standards in contributing to the development of higher quality guidelines in Australia. Currently, the inclusion of a consumer representative in guideline development groups is a NHMRC standard [21], but our findings are consistent with other research suggesting this approach may be of limited value when used in isolation and without proper support of consumers [18, 19]. Further clarification of consumer-engagement processes and their purpose could be driven by a revision of the NHMRC standards for clinical practice guidelines [21].

The limitations of this study need to be acknowledged. An extensive search was conducted, but it is possible that eligible guidelines were missed as, unlike other countries, such as the UK [41], there is no centralized guideline-development organization in Australia. The analysis conducted for this study was based on all publicly available information including published guidelines and their supporting documents. It is possible that more extensive consumer-engagement processes were conducted, but not reported. Since standardized quality-assessment practices, such as AGREE II, rely on published materials, there is a clear need for guideline developers to provide full information that accurately reports all elements of the development process.

## Conclusion

Clinical practice guidelines appear to be taking important steps towards supporting clinicians and patients through the incorporation of patient-preference recommendations, but there is scope for more explicit acknowledgement. Consumer-engagement processes used to develop guidelines have the potential to contribute to the identification of patient preferences, but further research is needed to investigate the contribution and impacts of these processes. Clarification of the consumer role and investment in consumer training may help to strengthen these processes and further support a systems-based approach to patient-centred care for people with multiple chronic conditions.

## Figures and Tables

**Table 1:** Appraisal of the included guidelines according to the patient-preference framework.

Guideline	Total core recommendations	PPR (% total)	PPR (ES)			
			Patient preferences	Care plans	Actively involved	Risks and benefits
Acute coronary syndromes [30]	44	2 (4.5)	1 (1)	1 (2)	0 (0)	0 (0)
Asthma [27]	158	3 (1.9)	0 (7)	3 (13)	0 (3)	0 (2)
Chronic heart failure [32]	80	1 (1.2)	1 (3)	0 (0)	0 (3)	0 (0)
Chronic kidney disease in type 2 diabetes^*^ [38]	18	0 (0)	0 (0)	0 (0)	0 (0)	0 (0)
Chronic obstructive pulmonary disease [37]	34	2 (5.9)	0 (4)	1 (6)	1 (0)	0 (0)
Coronary heart disease [31]	44	1 (2.3)	1 (0)	0 (3)	0 (0)	0 (1)
Early rheumatoid arthritis^*^ [35]	30	2 (6.7)	0 (3)	1 (4)	1 (1)	0 (0)
Hip and knee osteoarthritis^*^ [36]	34	2 (5.9)	0 (1)	2 (2)	0 (0)	0 (1)
Hypertension [29]	25	3 (12.0)	0 (2)	3 (5)	0 (0)	0 (0)
Locally advanced and metastatic prostate cancer [34]	57	7 (12.2)	2 (2)	0 (2)	0 (0)	5 (3)
Stroke^*^ [33]	335	16 (4.8)	1 (3)	13 (6)	0 (6)	2 (4)
Type 1 diabetes^*^ [25]	132	8 (6.0)	2 (3)	4 (3)	2 (0)	0 (0)
Type 2 diabetes [26]	85	2 (2.4)	0 (2)	1 (5)	1 (2)	0 (0)
Total	1,076	49 (4.5)	8 (31)	29 (51)	5 (15)	7 (11)

^*^Received National Health and Medical Research Council (NHMRC) approval. ES, evidence statements; PPR, patient-preference-related recommendations.

**Table 2:** Individual standardized Appraisal of Guidelines for Research and Evaluation (AGREE) II domain scores for the guidelines studied.

Guideline	Year	Scope and purpose (%)	Stakeholder involvement (%)	Rigour of development (%)	Clarity of presentation (%)	Applicability (%)	Editorial independence (%)
Acute coronary syndromes [30]	2006	67	58	19	69	25	33
Asthma [27]	2006	44	50	20	67	29	0
Chronic heart failure [32]	2011	53	50	14	72	13	79
Chronic kidney disease in type 2 diabetes^*^ [38]	2009	89	86	75	72	42	38
Chronic obstructive pulmonary disease [37]	2011	53	50	17	75	27	4
Coronary heart disease [31]	2012	31	39	11	69	27	63
Early rheumatoid arthritis^*^ [35]	2009	86	78	72	83	31	25
Hip and knee osteoarthritis^*^ [36]	2009	81	78	68	81	21	46
Hypertension [29]	2010	0	25	5	86	23	38
Locally advanced and metastatic prostate cancer [34]	2010	64	81	78	83	23	54
Stroke^*^ [33]	2010	83	86	74	78	71	88
Type 1 diabetes^*^ [25]	2011	92	81	76	78	54	46
Type 2 diabetes [26]	2012	53	47	8	72	40	0

%, overall score of the two reviewers calculated according to the AGREE II scoring system. Maximum AGREE II score 100%; COPD-X, chronic obstructive pulmonary disease and exacerbations.

^*^Received National Health and Medical Research Council (NHMRC) approval.

**Table 3:** Appraisal of the included guidelines according to the consumer-engagement framework.

Guideline	Consumers included in development group (n)	Role description	Provision of drafts for public review	Training and education
Acute coronary syndromes [30]	NR	NR	NR	NR
Asthma [27]	NR	NR	NR	NR
Chronic heart failure [32]	NI	NR	Yes	NR
Chronic kidney disease in type 2 diabetes^*^ [38]	5	Broad group and consumer specific	Yes	NR
Chronic obstructive pulmonary disease [37]	1	Broad group description	Yes	NR
Coronary heart disease [31]	NI	NR	Yes	NR
Early rheumatoid arthritis^*^ [35]	1	Broad group description	Yes	NR
Hip and knee osteoarthritis^*^ [36]	1	Broad group description	Yes	NR
Hypertension [29]	NI	NR	Yes	NR
Locally advanced and metastatic prostate cancer [34]	3	Broad group description	Yes	NR
Stroke^*^ [33]	2	Broad group description	Yes	NR
Type 1 diabetes^*^ [25]	2	Broad group description	Yes	NR
Type 2 diabetes [26]	NR	NR	NR	NR

^*^Received National Health and Medical Research Council (NHMRC) approval. NI, not included; NR, not reported.
